# A Nonimmunosuppressant Approach on Asia Psoriasis Subjects: 5-Year Followup and 11-Year Data Analysis

**DOI:** 10.1155/2012/304172

**Published:** 2012-01-11

**Authors:** Tony Yuqi Tang

**Affiliations:** Herose Clinical Centre, 190 Clemenceau Avenue, No. 03-24 Singapore Shopping Centre, Singapore 239924

## Abstract

Mono- or combine immunosuppressants are commonly used for psoriasis; however the side effect caused by potent systemic immunosuppressants frequently incurred; moreover the inflammation flares up shortly after immunosuppressants are discontinued. An alternative nonimmunosuppressive therapy was introduced to psoriasis subjects. A retrospective observational study consisted of 1583 psoriasis patients who were treated with Herose Psoria capsule 1440 mg three times daily at two clinical centres, one in China, the other in Singapore, from 1 January 2000 to 1 January 2011. Psoriasis lesion evolution was photographed at monthly visit, and efficacy and safety were assessed using psoriasis area severity index PASI score grading, renal and liver function testing, and adverse event reporting and supplemented by information obtained during targeted telephone interviews. The effectiveness of Herose on psoriasis was inversely associated to prior immunosuppressants exposure (*r* = 0.9154), significant improvements occurred in non-immunosuppressants subjects, and complete clearance was achieved in 8 months (87.5%, 14 of 16); the wavelike evolution of psoriatic lesion appeared in prior immunosuppressants subjects.

## 1. Introduction

Psoriasis is quite common; its prevalence ranges from 0.6% to 4.8% and shows a wide variability among ethnic groups [1]. The estimated prevalence of psoriasis is 0.123% in China, and there have been more than 3 million cases reported since 1984 [2]. In Singapore, psoriasis affects 1% to 2% of the population. Clinical manifestation of psoriasis is heterogeneous, ranging from limited to extensive. Guttate psoriasis is often a self-limited disease, lasting from 12 to 16 weeks without treatment [3]. It has been estimated that one-third to two-thirds of these patients later develop the chronic plaque type of psoriasis [4]; however, chronic plaque psoriasis is in most cases a lifelong disease and reported to wax and wane over time with episode of remissions and exacerbations [5], manifesting at unpredictable intervals. Erythrodermic and generalized pustular psoriasis have a poorer prognosis, with the disease tending to be severe and persistent [3]. 

Dermatological pathogenesis and medicine are ever-changing science. As new research and clinical experience broaden our knowledge, changes in treatment and drug therapy are required, previously psoriasis had been viewed as primarily a disease of hyperproliferation, and more recently it has come to be regarded as an autoimmune-mediated disease; hence the conventional systemic immunosuppressive (Corticosteroids, PUVA, MTX, cyclosporine) therapy started shifting to immune system partial (T cell, cytokines, chemokines, proteases, integrins) suppressive therapy, and alefacept and efalizumab targeted anti-inflammatory strategies are being tested with T-cell inhibitors. Adalimumab, certolizumab, etanercept, infliximab, and golimumab tumor necrosis factor (TNF) antagonists are approved; ustekinumab [6–8] and briakinumab [9] are inhibiting interleukins (IL) 12 and 23; clinical trials are underway of a few drugs that inhibit IL-17 and/or L-17 receptor. Another trial is studying an IL-22 blocking agent; meanwhile resveratrol, vb-201, apremilast, and tasocitinib are being studied as oral inhibitors of inflammatory cytokines.

The decision to implement therapy with immunosuppressants must be derived from a precise understanding of these agents and the often formidable adverse reactions that accompany their use (efalizumab which inhibit CD11a has been associated with PML progressive multifocal leukoencephalopathy [10]), for inflammatory and autoimmune-related skin disorders as psoriasis which is not life-threatening; it is incumbent on the treating physicians to determine that immunosuppressants are the appropriate form of treatment. Long-term remission and preventing recurrence were the priority in psoriasis management; this observational study was to describe the therapeutic response, tolerance, and safety of herose psoria capsule, a nonimmunosuppressive approach, in psoriasis patients.

## 2. Materials and Methods

We conducted a retrospective observational study and analysed 1583 consecutive psoriasis patients who were given Herose Psoria capsule at two clinical centres, Tang Jinghua Clinical Centre China and Herose Clinical Centre Singapore, between 1 January 2000 and 1 January 2011. The medical records and photodocumentation were reviewed.

To complete mean 5 years of continuous followup, we excluded patients who initialized herose psoria therapy after 1 January 2008; the patients who were lost to followup or dropped out for various reason were included; ultimately 1215 patients were included in this study. We collected the following information: age; psoriasis area severity index (PASI), prior psoriasis duration, dose and duration of prior treatment, comorbidities and concurrent treatments, therapeutic benefit, adverse events, and duration of followup.

Patients were informed that herose psoria [11, 12] capsule (HerosePharma, Singapore) (Table 1) was prescribed 1440 mg three times daily, and they gave written consent for its use. Treatment arm, herose psoria (Table 1) capsule is a botanical compound formulated to stimulate the yang component of the human body and was approved as a Chinese Proprietary Medicine (CPM) by Health Science Authority (HSA) of Singapore to treat psoriasis, manufactured by GMP factory in Singapore. Before starting herose, each patient underwent photograph taking, psoriasis area and severity index PASI grading; the blood test (liver function and renal function) was ordered for patients who were treated with systemic immunosuppressants previously.

During the study period, no other antipsoriatic treatment modalities, apart from herose psoria, were given, patients were advised to use topical Johnson & Johnson lotion or Vaseline to relieve skin dryness, antihistamine for itchiness, and antibiotics if required for secondary infection, psoriatic arthritis patiens were administrated with nonsteroid anti-inflammatory drugs (NSAIDs) by their rheumatologist, hypertension and hyperlipidaemia were given antihypertensive drugs and antihyperglycemic drugs by their cardioangiologist, diabetes and gastritis continued their antidiabetic medications and antigastritis or antiulcer agents, and asthma and eczema were advised non-steroids medications.

Patients discontinued immunosuppressants therapy 7 days before initiating herose and were clinically monitored on a monthly basis, digital photographs were taken at each follow-up visit, and physicians reviewed photographs taken before and after treatment, when available. The efficacy parameters included PASI with photograph comparison and the Physician Global Assessment (PGA) of the therapeutic response. The main criteria assessed were as follows: time to receive PASI 75% improvements; time to reach full clearance after starting heroes. Patients who received full clearance were reassessed for relapse via telephone followup annually till 1 January 2011. Adverse effects were assessed via clinical reporting, examination, lab studies.

Descriptive statistics were calculated using numbers with means, percentages, and range. To identify potential predictors of a good clinical response, we evaluated correlation coefficient between time to reach PASI75 improvement to cumulated previous immunosuppressants treatments, and psoriasis duration before herose treatment, and initial PASI score at herose treatment, and patients' age. Time serial psoriasis lesion evolution curve of both prior non-immunosuppressive treatment group and prior immunosuppressive treatment group was presented. Data were analysed and two-way graphs and matrix were produced by using STATA software (StataCorp, Tx).

## 3. Results and Discussion

**Figure 1:**
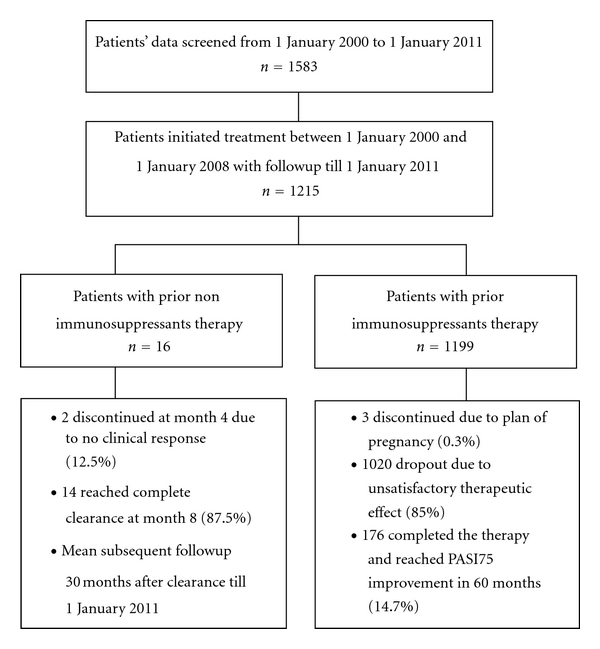
Flowchart and outcomes of the study.

A total of 1583 consecutive psoriasis medical records were collected at two clinical centers, one in China and the other in Singapore, between 1 January 2000 and 1 January 2011. To complete mean 5 years of continuous followup, ultimately 1215 patients were included in this study (Figure 1); of these patients with prior non-immunosuppressive therapy (*n* = 16), 14 patients (87.5%) reached complete clearance in 8 months thereafter with mean 30 months of subsequent followup; of those patients with prior immunosuppressive therapy (*n* = 1199), 176 patients (14.7%) completed therapy and achieved PASI75 in 60 months treatment. The main baseline characteristics and 60 months of screening of 1215 study subjects are summarized in Table 2. We found that 141 (11.6%), 103 (8.5%), 47 (3.9%), 123 (10.1%), 19 (1.6%), 28 (2.3%), 19 (1.6%), 18 (1.5%), and 18 (1.5%) of patients with psoriasis had comorbidities of hypertension, hyperlipidaemia, asthma, arthritis, hepatitis B, diabetes, gastritis, eczema, and gout, respectively, in which some of patients received more than one type of comorbidities. At the baseline, the average of patients' age is at 42.4 years (range, 18 years to 71 years), the average of psoriasis duration is 9.7 years with range from 1 year to 39 years, and the mean PASI is 28.9 with range from 3.6 to 68. Of the 1215 patients with psoriasis, only 16 (1.3%) received prior non-immunosuppressant therapy, and 1199 (98.7%) had previous immunosuppressive treatment. Table 2 reveals that 588 (48.4%) received less than 6 months of cumulated dose of prior immunosuppressants, and 332 (27.3%), 170 (14%), 33 (2.7%), and 76 (6.3%) received cumulated doses of prior immunosuppressants 6–12 months, 12–24 months, 24–36 months, and more than 36 months, respectively.

Table 3 shows the prior immunosuppressant modalities for psoriasis patients in this study, we also found that almost all patients with psoriasis had used topical steroids therapy (98.7%), and 46.6% had received systemic steroid (orally P.O. or intravenously I.V.) therapy. Majority of Asia psoriasis subjects had taken traditional herbal immunosuppressive decoction/powder medication (71.5%), and the commonly used herbal immunosuppressanst are listed in Table 3.

**Figure 2:**
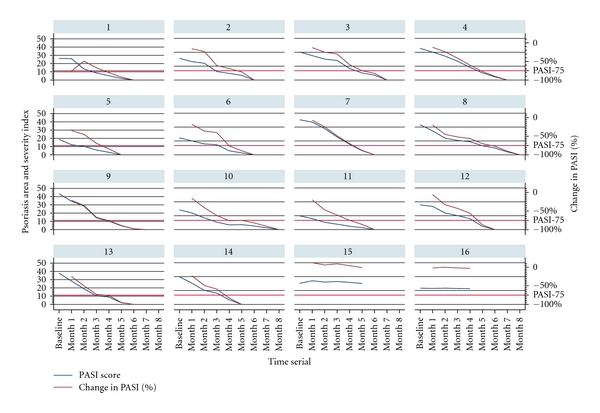
Psoriasis area and severity index scores and percentage change in PASI from the baseline screening to month 8 in 16 psoriasis patients without prior immunosuppressive therapy. After the start of herose, the severity of disease subsided gradually, 14 of 16 patients received complete clearance in 8 months, the mean treatment period to achieve PASI75 improvement is 4.5 months, and the mean subsequent follow-up period after clearance is 30 months; 2 of 16 patients had no clinical response and ceased the medication at month 4 and month 5, respectively.

**Figure 3:**
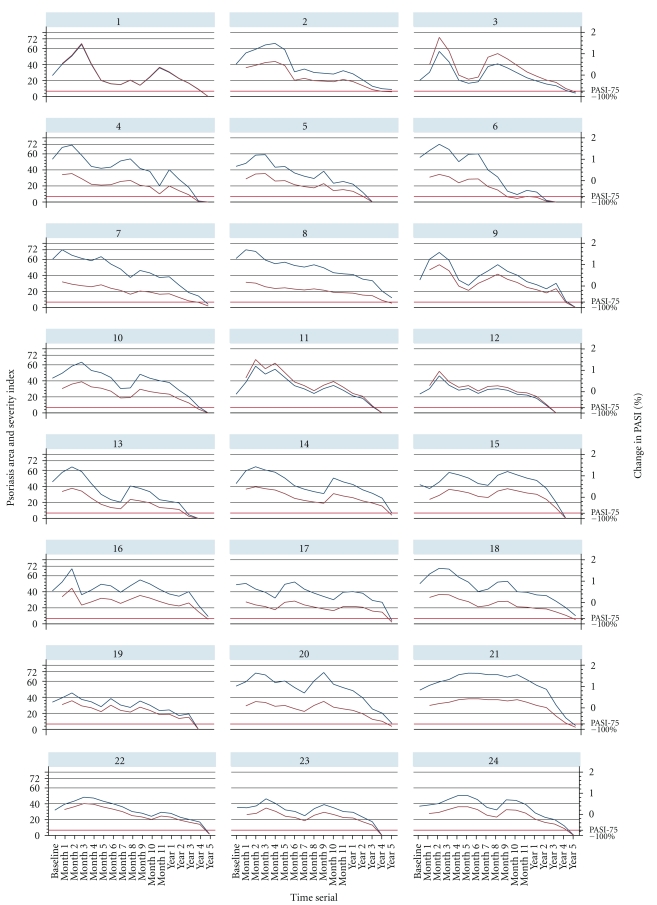

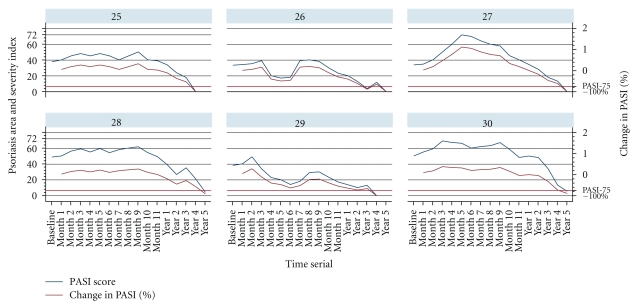
Psoriasis area and Severity index scores and percentage change in PASI from the baseline screening to month 60 in psoriasis patients with prior immunosuppressive therapy. 176 of 1199 patients with prior immunosuppressants therapy completed the study and reached PASI75 improvement in 60 months treatment and followup. The two-way graphs of 176 patients were generated, to save the space, a random selection of 30 of 176 patients was presented herewith on the above twoway graphs, patients underwent 7-day washouts of previous immunosuppressant treatments before starting herose, the severity of diseases flared up to a high point initially, and all patients (1199) experienced mean 110% (range from 55% to 240%) worsening of PASI score relative to baseline, but henceforth progressively tapered down when immunosuppressant withdrawal syndrome had subsided.

The patients with prior non-immunosuppressants therapy had shown significant response to herose, with 87.5% (14/16) experiencing PASI75 improvement at month 4.5, and received complete clearance in month 8 (Figure 2); in comparison with patients with prior immunosuppressants therapy, the absolute dropout rate is much lower at 12.5% (2/16) in 60-month study compared with prior immunosuppressants group which is 29% (347/1199), 57% (687/1199), 64% (764/1199), 72% (858/1199), 83% (995/1199), and 85% (1023/1199) at month 1, month 3, month 6, month 12, month 18, and month 60, respectively. All 1199 patients with prior immunosuppressants treatment experienced mean 110% (range, from 55% to 240%) worsening of PASI score relative to baseline value during the study (Figure 3), which was the immunosuppressants withdrawal syndrome [33–37] of psoriasis flare. At the end point, 14 (7.4%), 9 (4.5%), 1 (0.6%), 16 (8.5%), 1 (0.6%), and 1 (0.2%) psoriasis patients with comorbidities of hypertension, hyperlipidaemia, asthma, arthritis, hepatitis B, and eczema completed study and achieved Physician Global Assessment (PGA) score of “minimal” at month 60.

The C-reactive protein (CRP) and erythrocyte sedimentation rate (ESR) are often elevated in patients with psoriasis or psoriatic arthritis. Pertaining to the 176 patients with prior immunosuppressants treatment who completed this study, median CRP concentration and ESR are 1.5 mg/L and 24 mm/hr, respectively, at baseline. CRP concentrations declined during the 60-month study, with a median reduction from initial 1.5 mg/L to final value of 0.5 mg/L for psoriasis without arthritis (160/176) and 0.8 mg/L for psoriasis arthritis (16/176). ESR declined to normal range concurrently along with CRP, with final value of 11 mm/hr (age <50 years) and 15 mm/hr (age >50 years).

Elevated liver function test occurred in 231 patients with prior immunosuppressants therapy before initiating herose, median serum alanine aminotransferase (ALT) and aspartate aminotransferase (AST) levels were at 65 U/L and 56 U/L, respectively, at baseline, and it appears to be resulting from prior methotrexate therapy which had been associated with both acute and chronic hepatotoxicity. The median ALT and AST declined to 55 U/L and 48 U/L, 40 U/L and 30 U/L at month 5 and month 10, respectively, during the study. Elevation of blood urea nitrogen (BUN) and serum creatinine was observed in 65 patients with prior immunosuppressants therapy; it appears to be resulting from prior cyclosporine therapy, and it declined to 18 mg/dL and 1.0 mg/dL at month 5 after discontinue of the drug.

During the study, no systemic toxicities were uncovered by laboratory test, and no serious adverse effects were seen.

The enzymes 5-lipoxygenase and elastase are therapeutic targets in dermatological disorders such as psoriasis. Astragalus membranaceus inhibited 5-lipoxygenase with IC50 values of 141 microg mL(−1) [38]. The cellular immunity (T-lymphocyte subsets) study on one of herose ingredient astragali showed that the CD4 level and CD4/CD8 ratio increased levels of sIL-2R, and IgG and IgA lowered significantly [39]. Astragaloside Intravenous (ASI) increased T, B lymphocyte proliferation and antibody production *in vivo* and *in vitro*, but inhibited productions of IL-1 and TNF-alpha from peritoneal macrophages *in vitro* [40]. The haematology study of herose also showed that CD4 level increased by 20% and CD4/CD8 ratio increased by 20% in the immune-suppressed patient. The activation of nuclear transcription factor kappaB has now been linked with a variety of inflammatory diseases, including psoriasis and AIDS. Extensive research in the last few years has shown that the pathway that activates this transcription factor can be interrupted by phytochemicals ginger (gingerol) [41]. The zingiberene and zingerone, the extraction of ginger, promote adrenal medulla to double-release catecholamine and has marked anti-inflammatory effects [42, 43]. The *Cinnamomum* and *Codonopsis pilosula* have stimulatory effect on immunoglobulin (Ig) production by B cells and interleukin IL-1 production by monocytes *in vitro* [44]. Dansheng has action of promoting blood circulation and relieving blood stasis, resolving swelling [45]. The basic ingredient tanshinone can reduce aggregation of platelets, improve microcirculation and also has bacteriostatic and anti-inflammatory effects [46, 47]. White peony root lowers blood pressure and has anticonvulsion, anti-inflammation, and ulcer-prevention effects [48]; the *in vitro* testing has revealed that this herb possesses potent inhibitory activity against *Shigella dysenteriae*, *Salmonella typhi*, and *Staphylococcus aureaus*, and influenza virus to certain extent as well [47, 49]. The seeds of Jobstears act on spleen, stomach, and lung channels to remove dampness, tonify spleen, eliminate heat, and has sedative, analgesic, and antipyretic coixenolide effects [50]; the ethanol extraction of the herb is an effective therapy for Ehrlich ascites sarcoma and prolongs the lifespan of cancerous rats [51]. As the components of herose psoria do not suppress the immune system cells to achieve anti-inflammatory effect, it is unknown why non-immunosuppressant approach worked on psoriasis and why herose had a better effective therapeutic approach in the patients with prior non-immunosuppressants treatment than those with prior immunosuppressants.

Singapore Health Science Authority (HSA) required manufacturers with Good Manufacturing Practices (GMPs) to register herbal medicine and to license it as Chinese Proprietary Medicine (CPM) that complies with toxic heavy metals limits and microbial limits; companies need to carry out postmarketing surveillance and report serious adverse events. The Medicine Act and Regulations of Singapore require CPMs be properly labeled, free of adulterants, production controls, and appropriate documentation. The herbal medicine, like herose, uses plants extract to treat disease, either a single herb or mixture of different herbs. Unlike conventional drugs, in which the active substance is extracted from the chemicals, herbal medicines usually make use of the herb in its partial or whole form, herbs are complex substances with dozens or hundreds of chemical constituents, and often it is unclear which of these chemicals play an important role in the herb's pharmacologic activity; therefore determining which ingredient or ingredients should be considered active and thus would be subject to standardization can be difficult.

**Figure 4:**
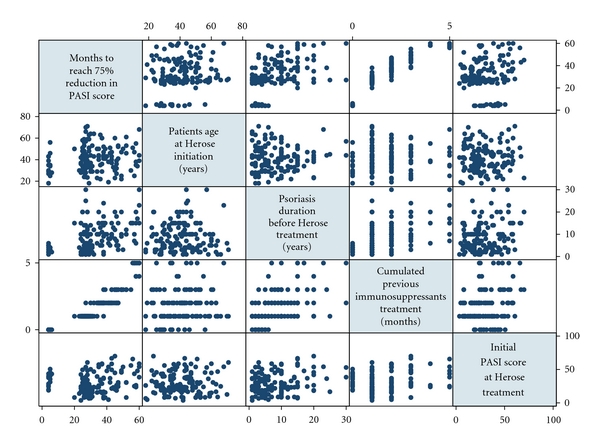
Correlation between months required reaching 75% reduction in PASI score and cumulated prior immunosuppressive treatment is strong (*r* = 0.9154) however the correlations to other factors, patients age, psoriasis duration, and initial PASI score, are weak (*r* = 0.1295, 0.4400, 0.1783, resp.). The time to reach PASI75 and clinical clearance significantly depends on the dosage and potency of the immunosuppressants that the patients had formerly consumed.

To establish potential predictors of a good clinical response with herose treatment, we tested correlation coefficient between months to achieve PASI75 and patients age, psoriasis duration, initial PASI score (Figure 4), and we found that months required reaching 75% reduction in PASI score are highly related with cumulated prior immunosuppressive treatment (*r* = 0.9154); however the correlations to other factors, patients age, psoriasis duration, and initial PASI score, are weak (*r* = 0.1295, 0.4400, 0.1783, resp.). The time to reach PASI75 and clinical clearance significantly depends on the dosage and potency of cumulated prior immunosuppressive therapy (Figure 4).

**Figure 5:**
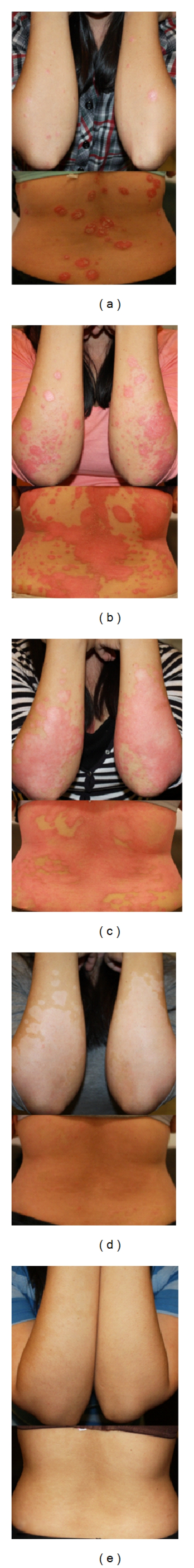
Progression of psoriasis improvement in patients with prior non-immunosuppressants therapy. Photographs taken before treatment, sharply marginated erythematous papule with a silvery-white scale (a). Existing psoriatic plaques developed and spread in sizes at month 1 (b). Flakes exfoliated from psoriatic lesion reduced in size, existing psoriatic plaques ceased further growth, and psoriatic plaques became thinner at month 2 (c). Existing psoriatic plaques eradicated and subsided lesion areas became paler than normal skin in color, similar to that in vitiligo. Notable improvement of PASI75 was achieved at month 3 (d). Skin areas with the vitiligo coloration took on a natural skin tone, and full clearance was received at month 5 (e).

**Figure 6:**
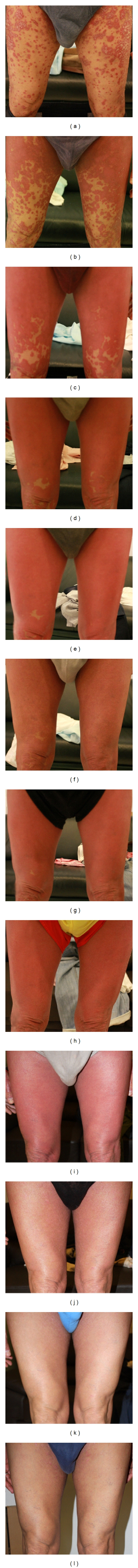
Evolution of psoriasis lesion in patients with prior immunosuppressants therapy. (a) Month 0. Photograph before initiating herose. (b) Month 1. (c) Month 2. (d) Month 3. (e) Month 4. (f) Month 5. (g) Month 6. (h) Month 9. (i) Month 12. (j) Month 18. (k) Month 26. (l) Month 32. Patient experienced 200% worsening of PASI score from month 1 (b) to month 6 (g) relative to baseline value (a), which was the immunosuppressants withdrawal syndrome, the severity of psoriasis flared up to a high point, but henceforth progressively tapered down, flakes exfoliated from psoriatic lesion reduced in size and finer flakes shed off, psoriatic plaques became thinner as shown at month 18 (j), improvement of PASI75 was achieved at month 26 (k), psoriatic plaques eradicated, and the subsided lesion area faded to a greyish colour (k, l).

During the study, we also observed that visible recovery began form head region downwards gradually spreading towards lower limbs in all responded patients with prior non-immunosuppressants therapy, complete clearance was within 8 months (Figure 2), when existing psoriatic plaques were eradicated, the subsided lesion areas became paler than normal skin color similar to vitiligo (Figure 5) compared to the normal surrounding skin, and the skin areas with vitiligo coloration evolved to a natural skin tone within three months without further medication (Figure 5). However in the patients with prior immunosuppressive therapy, we observed the improvement began from the lower limbs upwards gradually spreading towards upper trunks (Figure 6), or improvement began from the center of the psoriatic plaques with remission expanding towards the periphery of the psoriatic lesion; the subsided psoriatic plaques and subsided lesion area turn to a greyish color (Figure 6) rather than vitiligo color shown in patients in the prior non-immunosuppressants group (Figure 5). During the study, patients with prior immunosuppressant therapy likely started to experience the wavelike flare-up cycle (Figure 3). Symptoms of psoriasis which had previously been suppressed by immunotherapy appeared to resurface with vigor after the immune system is no longer being curtailed, and over the time, the flare-up subsided in frequency and intensity in a wavelike pattern (Figure 3). The time to reach the endpoint where the wave draws to a null appeared to depend on the dosage and potency of the immunosuppressants that the patients had formerly consumed (Figure 4). The interview with patients uncovered that the wavelike evolution of immunosuppressants withdrawal syndrome of psoriasis flare (Figure 3) is the most unsatisfied and challenging with them, which may explain the much higher dropout rate in the prior immunosuppressants group as shown in Table 2. If patients undergo lengthy washout period rather than 7-day washouts of prior immunosuppressants before initiating the herose therapy, it may help the reduction of frequency and intensity in wavelike flare-up pattern as shown in Figure 3, but such a transition strategy may be not practical as it requires a prolonged interval without therapy in psoriasis patients. In general practice, physicians may prefer to initiate an alternate therapy within a short time frame or an overlapping of therapies during transition to alternative.

## 4. Conclusions

The mechanism of action for clinical responses to herose, as well as its ability to modulate pathologic cellular and inflammatory pathways in skin lesions, needs to be investigated further, and this retrospective uncontrolled clinical observational study and previous clinical findings suggest that non-immunosuppressive approach may play an important part in management of immune system cell (T cell) to avoid misfiring and inappropriately targeting the skin cells; it may also be that pharmacogenomics is an important variable: there may be groups of people who have a better response to one or the other of therapeutic agents, perhaps because of polymorphisms in genes that control the expression of the relevant molecules. In our opinion, clinically useful conclusions can be drawn despite the limitations of this study design, we suggest the non-immunosuppressive approach may be used as the first-line therapy to improve the therapeutic outcome in patients with psoriasis, and further clinical investigation of controlled trial and mechanism of action is needed to be conducted for this indication.

## Figures and Tables

**Table 1:** Herose Psoria capsule* ingredients.

Ingredient	Amount (mg)
*Rhizoma Zingiberis *(Gan Jiang), ginger	429 mg
*Radix Paeoniae Alba* (Bai Shao), white peony root	156 mg
*Radix Astragali* (Huang Qi), astragalus root	313 mg
*Radix Salviae Miltiorrhizae* (Dan Shen), root of red-rooted salvia	556 mg
*Ramulus Cinnamomi* (Gui Zhi), cassia twig	299 mg
*Semen Coicis* (Yi Ren), Job's tears seed	538 mg
*Radix Codonopsis pilosula* (Dang Shen), root of *Codonopsis pilosula *	63 mg

*Each capsule consists of 360 mg extract equivalent to 2354 mg raw herbs.

**Table 2:** Baseline characteristics and follow-up data of the 1215 psoriasis patients with herose treatment.

	Baseline	Month 1	Month 3	Month 6	Month 12	Month 18	Month 60
	*n* = 1215	*n* = 868	*n* = 528	*n* = 449	n = 355	*n* = 218	*n* = 190
Age (yr) at herose initiation, mean (range)	42.4 (18–71)	41.7 (19–70)	41.7 (22–68)	42 (22–68)	41.7 (22–68)	41.9 (22–66)	41.5 (22–62)
Psoriasis duration (yr) before herose treatment, mean (range)	9.7 (1–39)	9.5 (1–39)	9.6 (1–39)	9.4 (1–39)	9.5 (1–39)	9.4 (1–25)	8.6 (1–25)
PASI score, mean (range)	28.9 (3.6–68)	39.7 (6.2–70)	45.2 (9.4–72)	38.4 (0–70)	29 (0–63.1)	32.7 (0–58.3)	1.4 (0–16.4)
Cumulated previous immunosuppressants treatment (mo), *n* (%)							
Null	16 (1.3%)	16 (1.8%)	16 (3%)	14 (3.1%)	14 (3.9%)	14 (6.4%)	14 (7.4%)
<6 months	588 (48.4%)	430 (49.5%)	268 (50.8%)	227 (50.6%)	164 (46.2%)	101 (46.3%)	95 (50%)
6–12 months	332 (27.3%)	231 (26.6%)	135 (25.6%)	116 (25.8%)	95 (26.8%)	54 (24.8%)	68 (35.8%)
12–24 months	170 (14%)	115 (13.2%)	79 (15%)	73 (16.3%)	63 (17.7%)	38 (17.4%)	13 (6.8%)
24–36 months	33 (2.7%)	27 (3.1%)	6 (1.1%)	0	0	0	0
>36 months	76 (6.3%)	49 (5.6%)	24 (4.5%)	19 (4.2%)	19 (5.4%)	11 (5.1%)	0
Past illness, percentage							
Hypertension	141 (11.6%)						14 (7.4%)
Hyperlipidemia	103 (8.5%)						9 (4.5%)
Asthma	47 (3.9%)						1 (0.6%)
Arthritis	123 (10.1%)						16 (8.5%)
Hepetitis B	19 (1.6%)						1 (0.6%)
Diabetes	28 (2.3%)						0
Gastritis	19 (1.6%)						0
Eczema	18 (1.5%)						1 (0.6%)
Gout	18 (1.5%)						0

**Table 3:** Immunosuppressants modalities in China and Singapore patients with psoriasis.

Immunosuppressants modalities in China and Singapore patients with psoriasis	%
Topical	
Corticosteroids	98.7%
Calcipotriol	30.2%
Retinoid	11.4%
Tacrolimus	3.8%
Systemic	
Systemic glucocorticoid	46.6%
UVB or PUVA	21.5%
Methotrexate	15.7%
Cyclosporine	5.2%
Azathioprine	0.2%
Hydroxyurea	0.2%
Herbal decoction	
*Glycyrrhiza* (Gan Cao), Licorice [13–15]	71.5%
*Lonicera* (Jin Yin Hua), Honeysuckle	71.5%
*Dictamnus* (Bai Xian Pi), Burning Bush [16]	71.5%
*Cryptotympana *(Chan Tui), Cicada Slough	71.5%
*Tripterygium* (Lei Gong Teng), Threewingnut [17–20]	14.5%
*Sophora* (Ku Shen), Flavescent Sophora [15, 21, 22]	62.4%
*Prunella* (Xia Ku Cao), Self Heal [23]	71.5%
*Curcuma* (Jiang Huang, Yu Jin), Turmeric [24–27]	71.5%
*Bupleurum* (Chai Hu), Thorowax [28–30]	23.0%
*Cordyceps sinensis* (Dong Chong Xia Cao) [31, 32]	35.4%
